# You see what you look for: Targets and distractors in visual search can cause opposing serial dependencies

**DOI:** 10.1167/jov.21.10.3

**Published:** 2021-09-01

**Authors:** Mohsen Rafiei, Andrey Chetverikov, Sabrina Hansmann-Roth, Árni Kristjánsson

**Affiliations:** 1 Icelandic Vision Lab, Faculty of Psychology, University of Iceland, Reykjavík, Iceland; 2 Donders Institute for Brain, Cognition and Behavior, Radboud University, Nijmegen, Netherlands; 3 Icelandic Vision Lab, Faculty of Psychology, University of Iceland, Reykjavík, Iceland; 4 Sciences Cognitives et Sciences Affectives (SCALab), Université de Lille, Lille, France; 5 Icelandic Vision Lab, Faculty of Psychology, University of Iceland, Reykjavík, Iceland; 6 School of Psychology, National Research University, Higher School of Economics, Moscow, Russian Federation

**Keywords:** visual search, perceptual bias, visual attention

## Abstract

Visual perception is, at any given moment, strongly influenced by its temporal context—what stimuli have recently been perceived and in what surroundings. We have previously shown that to-be-ignored items produce a bias upon subsequent perceptual decisions that acts in parallel with other biases induced by attended items. However, our previous investigations were confined to biases upon the perceived orientation of a visual search target, and it is unclear whether these biases influence perceptual decisions in a more general sense. Here, we test whether the biases from visual search targets and distractors affect the perceived orientation of a neutral test line, one that is neither a target nor a distractor. To do so, we asked participants to search for an oddly oriented line among distractors and report its location for a few trials and next presented a test line irrelevant to the search task. Participants were asked to report the orientation of the test line. Our results indicate that in tasks involving visual search, targets induce a positive bias upon a neutral test line if their orientations are similar, whereas distractors produce an attractive bias for similar test lines and a repulsive bias if the orientations of the test line and the average orientation of the distractors are far apart in feature space. In sum, our results show that both attentional role and proximity in feature space between previous and current stimuli determine the direction of biases in perceptual decisions.

## Introduction

Our visual system needs to process a large amount of complex visual information at any given moment. To make this task easier, the brain uses various heuristics based on knowledge about the environment. For example, we know that the appearance of an object typically does not change dramatically from one moment to the next. This means that our visual system may ignore negligible changes in the visual input to promote stability. However, when objects do indeed change, the same heuristic might lead to biases. One example of this is serial dependence (e.g., Fischer & Whitney, 2014; Pascucci, Mancuso, Santandrea, Della Libera, Plomp, & Chelazzi, 2019). In Fischer and Whitney (2014), observers viewed an inducer line, followed by an oriented line whose orientation had to be reported. They found that orientation estimates for this second line were biased toward the inducer orientation. They concluded that perception is tuned toward previous stimuli that have similar features and appear in the same locations and proposed that serial dependence promotes perceptual stability in the visual environment (for reviews, see Burr & Cicchini, 2014; Cicchini & Kristjánsson, 2015; Kiyonaga, Scimeca, Bliss, & Whitney, 2017). Further investigations have since revealed that the perception of many other features, such as shape (Manassi, Kristjánsson & Whitney, 2019), motion coherence (Suarez-Pinilla, Seth, & Roseboom, 2018), numerosity (Fornaciai & Park, 2018), facial identity (Liberman, Fischer & Whitney, 2014), and even stimulus ensembles (Manassi, Liberman, Chaney, & Whitney, 2017; Pascucci et al., 2019), is systematically biased by information from the recent past.

Serial dependence in perception is thought to help us keep perception stable against minor changes that might arise due to internal or external noise. But, the stimuli we encounter are not all equally important, and some can be ignored to enable us to concentrate on the object of interest at a given moment. For example, during visual search we need to pay attention to items similar to the potential target while simultaneously ignoring stimuli dissimilar to the target. This raises the question of whether and how these dissimilar items that need to be ignored affect our perceptual decisions.^1^

Fritsche and colleagues (Fritsche & de Lange, 2019; Fritsche, Mostert, & de Lange, 2017; see also earlier work reviewed by Hsu, 2021) have suggested that proximity in feature space between the test stimulus and the inducer may determine whether biases from serial dependence are repulsive or attractive. According to Fritsche et al., an attractive orientation bias occurs when preceding targets and/or distractors have similar orientations. In contrast, a repulsive bias occurs when they have dissimilar orientations.

In a recent paper, we studied the effect of distractors upon perceptual decisions about the attended items (targets) during visual search for an oddly oriented line among distractors (Rafiei, Hansmann-Roth, Whitney, Kristjánsson, & Chetverikov, 2021). In visual search, observers can surprisingly quickly learn the probability distributions of distractor sets (Chetverikov, Campana & Kristjánsson, 2016; Chetverikov, Campana & Kristjánsson, 2017a; Chetverikov, Campana & Kristjánsson, 2017b; Chetverikov, Campana & Kristjánsson, 2017c; Chetverikov, Campana & Kristjánsson, 2017d; Chetverikov, Campana & Kristjánsson, 2020a; Hansmann-Roth, Chetverikov, & Kristjánsson, 2019; Hansmann-Roth, Kristjánsson, & Chetverikov, 2020a; Hansmann-Roth, Kristjánsson, Whitney, & Chetverikov, 2021; Tanrıkulu, Chetverikov & Kristjánsson, 2020). They can learn which distractor features are more probable than others in surprising detail, and, importantly, unlike the items typically used in serial dependence studies, observers learn to ignore them. Following this approach, in Rafiei et al. (2021) we employed repeated distractor presentations over several trials to ensure that participants learn the distractor features while judging the location of an oddly oriented target. After a few search trials, participants were asked to report the target orientation on the last visual search trial. We found that the perceived orientation of the target was *pushed away* from the mean orientation of the distractors. Additionally, the search targets induced an *attractive* bias upon the perceived orientation of a subsequent visual search target, a result in line with serial dependence findings. Our study demonstrated that the search task creates conditions for two simultaneous perceptual biases: a repulsive bias from distractors and an attractive bias from targets.

Although our findings (Rafiei et al., 2021) show how to-be-ignored items produce a perceptual bias that acts in parallel with another bias induced by attended items, our investigation was confined to biases upon the perceived orientation of the visual search target. We did not address whether the biases influence perceptual decisions more broadly. Here, we address the question of whether the biases from visual search targets and to-be-ignored distractors reported by Rafiei et al. (2021) can alter perceptual processing in a more general sense, or specifically whether the biases affect the perceived orientation of a neutral test line that was neither a target nor a distractor. To do so, we asked our participants to search for an oddly oriented line among distractors and report its location for several adjacent trials. The specific targets and distractors varied from trial to trial, but their respective probability distributions remained stable within each block of search trials to ensure that the distractor feature distribution and the targets were well encoded. Next, participants were asked to report the orientation of a briefly presented test line in an adjustment task. We aimed to assess the biases induced by targets and distractors on the perceived orientation of the test line that was, crucially, unrelated to the visual search task.

Rafiei et al. (2021) proposed that the roles the stimuli in the visual field play in attentional tasks determines whether any biases from presented stimuli are attractive or repulsive. They suggested that to-be-ignored objects (such as distractors) lead to repulsive biases upon the perceived orientation of the target, whereas attended stimuli (such as the previous targets) yield attractive biases upon subsequent perceptual decisions. In Experiment 1, we tested whether similar effects would occur for a task-irrelevant line. The distance in feature space (orientation) between the target and distractors, on the one hand, and the test line, on the other, was random. In Experiments 2 and 3, we therefore addressed the role of distance in feature space between the test line on the one hand and the target and distractors on the other more systematically in light of the findings of Fritsche et al. (2017) and Fritsche and de Lange (2019). Finally, in Experiment 4, we tested the biases induced by neutral stimuli (which are neither search targets nor distractors). We cued the target location while keeping the task the same in all other aspects so that participants did not need to search for the target. Therefore, the lines around the cued line did not serve as distractors anymore but were neutral within the task. If their role as distractors is crucial for determining the direction of the biases, the biases should be eliminated or strongly diminished when the search is no longer required.

In sum, we had three aims in the current project. In Experiment 1, we studied biases produced by visual search upon a neutral test object. In Experiments 2 and 3, we investigated the effect that distance in feature space between the visual search targets and distractors and the task-irrelevant test line has on these biases. Finally, in Experiment 4, we tested how cueing the target location (presumably eliminating the need for a search) would affect the biases from targets and distractors in the display upon the perceived orientation of the task-irrelevant test line.

## Experiment 1

**Figure 1.**
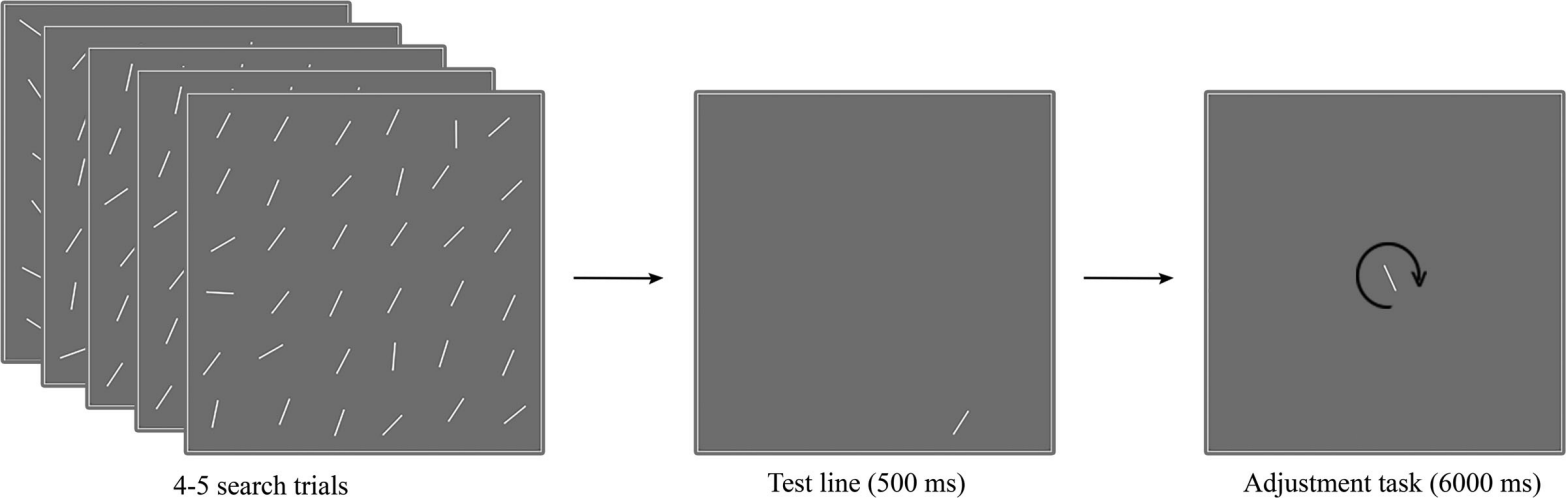
The design of Experiment 1. The figure shows one block consisting of the search display, the task-irrelevant test line, and the adjustment task. First, participants were required to complete four or five visual search trials. They searched for an oddly oriented line (in the example shown here, the target of the last trial is located in the first column, fourth row) in the search array of 36 lines displayed in a 6 × 6 matrix. Next, a quasi-randomly oriented line (test line) was shown at a quasi-randomly chosen location. Finally, participants had to report the perceived test line orientation by adjusting a single bar presented at the screen center.

In Experiment 1, we tested whether the orientation of a target and distractors in a visual search task leads to biases upon perceptual decisions about the orientation of a task-independent test line presented following a series of visual search trials. In each block, participants were asked to perform a series of visual search trials (learning trials) to ensure that they had a representation of distractors, as in studies involving the feature distribution learning method (Chetverikov et al., 2016; Chetverikov, Hansmann-Roth, Tanrikulu, & Kristjánsson, 2020b). Next, a randomly oriented test line was shown on the screen for 500 ms. Finally, participants had to report the test orientation of the test line by adjusting a subsequently presented line located at screen center (see Figure 1).

### Method

#### Participants

Twenty participants (11 females and nine males; mean age = 32.35 years) were recruited for Experiment 1. All participants had normal or corrected-to-normal vision and provided written informed consent that described the experimental procedure before starting the study. For all of the experiments here, before starting the test sessions, any participants who had never participated in our similar experiments underwent a training session, which was similar to the test session with the same number of experimental blocks. After completing the training session, participants performed the test trials. The training and test sessions were held on two different days.

#### Stimuli and procedure

The stimuli were displayed at a viewing distance of 70 cm on a 24-inch Asus (Taipei, Taiwan) monitor with a resolution of 1920 × 1080 pixels. The experiment was programmed and carried out using Psychophysics Toolbox Version 3 (Brainard, 1997; Kleiner, Brainard, & Pelli, 2007) in MATLAB 2016a (MathWorks, Natick, MA). We employed the feature distribution learning method (Chetverikov et al., 2016), where participants were asked to complete four or five visual search trials in each experimental block to ensure that they had learned the distractor distribution. On these visual search trials, participants searched for an oddly oriented line in the center of the screen in an array of 36 white lines (length = 1° of visual angle), arranged in a 6 × 6 matrix (16° × 16° at the center of a screen) on a gray background. We randomly added ±0.5° to both the vertical and horizontal coordinates of the line positions to introduce some irregularity to the search array. If the target was in the upper three rows, participants were required to press the “E” key (on a standard keyboard) and the “D” key when the target was in the lower three rows (see Figure 1).

We used both feedback and a scoring system to encourage participants to respond as quickly and accurately as possible on the search trials. If the provided response was incorrect, the word “Error” appeared in red on the screen for 1 second. The score on the last trial was presented in the top-left corner of the screen during the search trials, and a cumulative score was shown during the breaks. We employed the following formula to calculate the scores for correct answers: score = 10 + (1 – RT) × 10, where RT stands for the response time in seconds, and the following equation determined the scores when responses were incorrect: score = –[10 + (1 – RT) × 10] – 10. If the given response was correct and made in less than 2 seconds, the score was positive; otherwise, the score was negative.

After completing the search trials, the test line (a single oriented line) was presented on the screen for 500 ms. In half of the blocks, the test line was shown at the last search target position; in the rest of the blocks, it was displayed at a randomly chosen distractor position. The participants were asked to report the test line orientation by adjusting a bar located in the middle of the screen. Participants had 6 seconds to press the “M” or “N” keys to rotate the adjustment line clockwise or counterclockwise, respectively.

The mean distractor orientation on search trials was selected randomly for each block. The distractors were taken from a Gaussian distribution with a standard deviation of 15° or a uniform distribution with a range of 60° (the distribution type remained constant within a block; its effect is not analyzed here). Within each block, the distractor distribution mean was kept constant to allow observers to learn the distractor distribution (as shown in previous experiments; for a review, see Chetverikov et al., 2020b). The target orientation was selected pseudorandomly for each trial within 60° to 120° relative to the mean of the distractor distribution.

**Figure 2.**
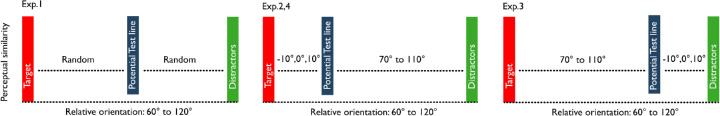
Proximity in feature space of test line, distractors, and target orientation in the experiments. In Experiment 1, the distances in feature space between the test line and target and between the test line and distractors were selected randomly. In Experiments 2 and 4, the test line was close to the target and far away from the distractors. In Experiment 3, the test line was close to distractors and far from the target.

As shown in Figure 2, the distances in orientation space between the test line and the last search target and the test line and the distractor mean were selected randomly (so the test line orientation was also selected randomly). Accordingly, in half of the blocks, the test line orientation was clockwise relative to the mean orientation of the distractors and counterclockwise in the rest of the blocks. Similarly, the test line was clockwise relative to the target on half of the trials and counterclockwise otherwise.

### General data analysis

We excluded blocks with incorrect answers on the last search trial to ensure that we only investigated blocks where we could be reasonably sure that participants had learned the orientation of the target and the distractor distribution. So, in Experiment 1, 313 blocks (6.13% of all of the blocks), 501 blocks (6.32%) in Experiment 2, 524 blocks (6.61%) in Experiment 3, and 860 blocks (8.14%) in Experiment 4 were excluded from the data before analyses. To estimate the effects of the previous target and distractor on the test line orientation judgment, we employed a hierarchical Bayesian model that integrates all of the participants’ data in a single model and accounts for the uncertainty of parameter estimates. The model consisted of a mixture of two distributions of behavioral responses (*x*), each reflecting different types of responses on the adjustment task. The Gaussian distribution, with probability density *f_N_*(*x*; *µ*, *σ*^2^), represents variability and biases in adjustment errors; the uniform distribution, spanning orientation space with probability density *f_U_*(*x*) = 1/180, maps the participants’ random guesses (Zhang & Luck, 2008). The two distributions are mixed with the λ probability of an observation coming from a Gaussian distribution:

$$\begin{array}{c} f\left(x;\theta ,\mu ,\sigma^{2}\right)=\lambda f_{N}\left(x;\mu ,\sigma^{2}\right)+\left(1-\lambda \right)f_{U}\left(x\right). \end{array}$$

Note that the Gaussian distribution is used here because the errors were relatively small so that the circularity of orientation space was not a concern.

We modeled the mean of the Gaussian distribution (systematic biases) with a Bayesian hierarchical linear model as a function of the relationship between the distractors and the test line (clockwise vs. counterclockwise; in the later experiments, we also added “no difference” or “orthogonal” conditions to the model as dictated by the experimental design) and the target to the test line relationship (clockwise vs. counterclockwise; again, in the later experiments, we added “no difference” or “orthogonal” conditions where appropriate) as fixed effects. The differences among participants in terms of the overall mean error (the intercept in the model), the effects of targets and distractors (the slopes in the model), and the mixture proportions (λ) were modeled as random effects.

Furthermore, to test how much the results depend on using the Zhang and Luck model (mixture of Gaussian and uniform), we repeated the analyses using a simple repeated-measures analysis of variance (ANOVA), in which the adjustment error was the dependent variable, and the distractor to test line conditions and the target to test line conditions were the independent variables. The results were almost identical (check the Supplementary Material for more detail).

### Results and discussion

Observers’ visual search performance followed the expected pattern. Response times (*M* = 895 ms, *SD* = 270) decreased within the block, F(4, 76) = 18.52, p < 0.001, η^2^*_G_* = 0.02, whereas accuracy (*M* = 94.0% correct, *SD* = 3.3) remained relatively constant, F(4, 76) = 0.79, p = 0.494, η^2^*_G_* = 0.01, reflecting a typical attentional priming effect (Kristjánsson & Ásgeirsson, 2019). This suggests that observes obtained information about probable target and distractor features during the search.

**Figure 3.**
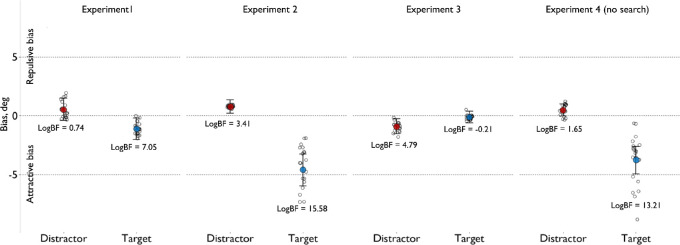
The target and distractor effects on adjustment error in the reported test line orientation for Experiments 1 to 4. Small gray dots represent the individual observers, and large colored dots represent the population-level effects. The lines display 95% credibility intervals. Effect estimates (*y*-axis) show the magnitude of the biases (in degrees) produced by distractors and targets, and the *x*-axis shows the sources of the biases (distractors and targets).

We then analyzed the role of observed distractors and targets on the judgments of the orientation of an independent test line. In the adjustment task, observers were relatively precise (*M* = −0.004°, *SD* = 12.16°). As shown in Figure 3, the previous target had an attractive effect (*b* = −1.08; 95% HPDI = −2.01 to −0.14, where HPDI denotes the highest posterior density interval, a form of credibility interval defining the plausible range within which the unobserved parameter might vary) and the distractor effect was numerically repulsive (*b* = 0.54; 95% HPDI = −0.43 to 1.51). To further test the effect of distractors and the target, we compared the full model with the restricted distractors-only (dropping the target effect) and target-only (dropping the effect of the distractors) models. The full model provided a better fit than both the distractors-only (logBF = 7.05, where logBF stands for log-transformed Bayes factor with positive values, which here indicates evidence in favor of the full model) and target-only models (logBF = 0.74). So, as seen before in Rafiei et al. (2021), the distractor sets led to a repulsive serial dependence effect and the target caused an attractive effect upon the perceived orientation of the test line. Importantly here, these biases were observed for a task-irrelevant test line that was neither a target nor a distractor. However, the credibility interval for the distractor effect includes zero, and the logBF factor for the target-only model is small, indicating that we cannot draw strong conclusions from it.

Additionally, we ran an exploratory analysis of target- and distractor-to-test distances as continuous variables without splitting trials into clockwise/counter-clockwise groups (shown in Supplementary Fig. S1). The results suggest that the target effect was similar to what we observed previously (Rafiei et al., 2021), with positive biases being created by test lines relatively similar to the targets and no bias from test lines dissimilar to the targets. For distractors, in contrast, the biases were repulsive and became stronger with decreasing similarity. However, due to the nature of the task, the orientations of targets and distractors were not fully independent, and therefore also the effect of their similarity to the test line (the target must be dissimilar to distractors). Therefore, we treated this analysis as exploratory and further addressed the tested effects of similarity in the following experiments.

## Experiments 2 and 3

The results of Experiment 1 indicate that, although to-be-ignored objects (in our case, distractors during visual search) led to repulsive serial dependence effects upon perceptual decisions, the attended items (targets) caused an attractive bias. Importantly, this occurred not only for visual search targets but also for a task-irrelevant test line, indicating that this is not simply a task-based bias but causes general biases upon perceptual decisions. Yet, the evidence for the distractor effect was not significant. In Experiments 2 and 3, we looked at proximity in feature space as a potential moderating factor for both target and distractor effects.

Some recent studies have shown that proximity in feature space between what we have recently perceived and what we are currently observing can determine the direction of serial dependence produced by preceding items (whether the biases are attractive or repulsive). Fritsche et al. (2017) showed that two stimuli could induce opposite biases, depending on their distances in feature space. In Experiments 2 and 3, we therefore manipulated the distances in feature space between the distractors and test line and between the target and the test line to investigate the effect of proximity in feature space on the biases produced by our visual search stimuli (Figure 2).

### Method

#### Participants

Twenty participants (13 females and seven males, mean age = 31.3 years for Experiment 2; 17 females and three males, mean age = 28 years for Experiment 3) were recruited. All had normal or corrected-to-normal vision and provided written informed consent before starting the tests which briefly explained the experimental procedure.

#### Stimuli and procedure

The methods in Experiments 2 and 3 were overall similar to those for Experiment 1. In Experiment 2, the test line orientation was close to the target orientation and far away from the mean of the distractor distribution. The mean distractor orientation for each block was picked randomly (from 0° to 180°), and the test line orientation was selected so that it ranged from 70° to 110° (in 4° steps) away from the distractor distribution mean with an equal number of trials within each distance bin. On the last visual search trial within each block, the target orientation had a 10°, 0°, or −10° distance to the test line (counterbalanced). On trials preceding this last trial, the target was selected from a uniform distribution with 60° to 120° distances from the distractor mean. So, to ensure that the biases produced by the target and distractors were not confounded, all of the distances in feature space between the test line orientation and the mean distractor orientation and target orientation were counterbalanced.

Because our aim was to address the role of relations in feature space between targets and distractors, on the one hand, and the test line, on the other, in Experiment 3, in contrast with Experiment 2, the test line orientation was close to the mean of the distractors and far from the target (Figure 2). The mean distractor orientation was selected randomly from 0° to 180°, as in Experiment 2. Next, the test line orientation was picked from 10°, 0°, or −10° distances to distractors. The distractors were, therefore, close to the test line in feature space. The target orientation was also chosen from 70° to 110° (in 4° steps) from the test line orientation.

### Results and discussion

In both Experiments 2 and 3, priming effects were observed, suggesting that observers learned target and distractor characteristics within each block. In Experiment 2, the RT, F(4, 76) = 6.11, p = 0.016, η^2^*_G_* = 0.02, *M* = 825, *SD* = 200, decreased and accuracy, F(4, 76) = 2.94, p = 0.045, η^2^*_G_* = 0.02, *M* = 93.4, *SD* = 3.9, increased significantly over the visual search trials. In Experiment 3, the priming effects for accuracy, F(4, 76) = 3.66, p = 0.015, η^2^*_G_* = 0.01, *M* = 92.7, *SD* = 4.5, and RT were also significant, F(4, 76) = 9.41, p = 0.002, η^2^*_G_* = 0.02, *M* = 729, *SD* = 160).

The target and distractor effects on adjustment error for Experiments 2 and 3 are shown in Figure 3. Overall, the adjustment error was similar to that for Experiment 1 (*M* = 0.17°, *SD* = 14.28° for Experiment 2; *M* = 0.004°, *SD* = 10.38° for Experiment 3). Both attention and proximity in feature space between the inducers (targets and distractors) and the test line clearly affected the direction and magnitude of the serial dependence effects (Figure 3). In Experiment 2, the targets (close to the test line in feature space) caused an attractive bias (b = −4.61; 95% HPDI = −5.96 to −3.22), and the distractors (far away from the test line) caused a repulsive bias (b = 0.78; 95% HPDI = 0.24–1.35). Comparing the restricted models (dropping the target or distractor effect) against the full model, we found that the full model provided a better fit in both comparisons (full model vs. target-only, logBF = 3.41; full model vs. distractors-only, logBF = 15.58).

In contrast with Experiment 2, in Experiment 3, where the test line was similar to distractors and differed from targets, the direction of serial dependence for distractors was reversed—the distractors induced an attractive bias (b = −0.92; 95% HPDI = −1.56 to −0.27), and the target-induced bias was close to zero (b = −0.12; 95% HPDI = −0.63 to 0.39). The full model provided a slightly worse fit than the distractors-only model (logBF = −0.21) but predicted the data better than the target-only model (logBF = 4.79). Therefore, the results for Experiment 3 indicate, in contrast with Experiment 2, that the distractors played a larger role in shaping the adjustment error than the targets and created attractive and not repulsive biases.

Overall, the results of Experiments 2 and 3 show that proximity in feature space between what we have already perceived and what we observe determines the direction of the biases from visual search distractors and targets. This means that attention (or whether an item is a target or distractor) is not the only factor determining the direction of the biases. In Experiment 2, the targets induced an attractive bias and the distractors a repulsive bias (as in Experiment 1), whereas in Experiment 3 this was reversed; the distractors produced an attractive bias upon perceptual decisions of the orientation of the test line even though they were to be ignored. On the other hand, the attended stimuli (the targets) did not affect the perceived orientation of the test line. Therefore, Experiments 2 and 3 argue strongly that feature space proximity plays a large role in determining bias direction.

## Experiment 4

The results of Rafiei et al. (2021) showed how attention plays a role in shaping biases from serial dependence. Distractors that must be ignored led to a repulsive bias, and attended targets introduced attractive biases. This conclusion was supported in Experiments 1 and 2 here. However, the results of Experiment 3 complicate this story because they show that proximity in feature space between what we have perceived previously (targets or distractors) and what we currently perceive modulates the direction of the biases. In Experiment 4, we aimed to assess the role of attention in forming perceptual biases by converting the distractors from to-be-ignored stimuli to neutral ones by cueing the target location.

### Method

#### Participants

As in the preceding experiments, we recruited 20 participants (12 females and eight males; mean age = 30.95 years). All had normal or corrected-to-normal vision and signed informed consent where the experimental procedure was outlined briefly.

#### Stimuli and procedure

In Experiment 4, the methods were similar to those of Experiment 2, where the targets were close to the test line orientation and the distractors were far from it. However, in this experiment, the crucial difference was that the target location was cued by a small dot presented for a short period (500 ms) before the visual search trial started. The size of the light-gray dot was 3 pixels, and it was shown 30 pixels (0.54° visual angle) above or below the target line center for 500 ms. We reasoned that if participants were cued to the target location, they would not need to search for the target among the distractor lines, which would therefore not have to be actively rejected as nontargets. The task was to report the target position relative to the cueing dot, so participants were to press the “D” key if the target appeared below the cue and “E” if the target appeared above it. After completing four or five such trials in each block, an irrelevant test line was presented, followed by the adjustment line like in previous experiments.

### Results and discussion

In Experiment 4, adjustment errors were similar in magnitude to those of previous experiments (M = 0.25°, SD = 9.93°). The targets produced an attractive bias in the perceived orientation of the test line (*b* = −3.76; 95% HPDI = −4.89 to −2.57) (see the plot for Experiment 4 in Figure 3). In contrast, the effect of distractors was repulsive but close to zero (*b* = 0.48, 95% HPDI = −0.02 to 1.01). The model comparisons showed that the full model, which included both effects, fit the data better than both the distractors-only model (logBF = 13.21) and targets-only model (logBF = 1.65).

The results of Experiment 4 suggest that the role of proximity in feature space may be just as important than the role of attention. When the distractors were converted to “neutral” stimuli with a pre-cue, the distractors still produced a repulsive bias in perceived test line orientation. We speculate that parts of the biases that we see reflect stimulus-based, not attentional, factors; in other words, even though the distractors did not play a distracting role, they nevertheless biased subsequent perceptual decisions through merely being present on the screen.

## General discussion

In Rafiei et al. (2021), we demonstrated for the first time, to our knowledge, how attended and ignored stimuli in visual search create perceptual biases. We argued that at least two opposite biases influence perceptual decisions of a search target. Positive serial dependence pulls the target toward previous target features, and a negative bias pushes targets away from distractors. Here, we set out to address three questions regarding biases created by targets and distractors during visual search, this time upon perceptual decisions of a neutral test object. Our main conclusions are the following:

1. There were biases from both preceding targets and distractors upon perceptual decisions of a neutral, task-irrelevant test line. Overall, attended items (targets) produced stronger serial dependence than ignored ones (distractors).
2. Both attention and proximity in feature space played important roles in determining the perceptual biases from serial dependence.
3. We tested how cueing the target location (presumably eliminating the need for search) affected serial dependence biases. Even when the distractors were not “to-be-rejected” items anymore but were irrelevant to the task (and dissimilar to the test item), they still produced repulsive biases. These results show that even if their attentional role is weakened, distractors can still cause biases, arguing for a lower-level bias from the repeated distractors.

### What functional role do the biases play in perceptual decisions?

The first thing to note is that the current results show that serial dependence biases from visual search operate on perceptual decisions generally, not just on the search-relevant items. Rafiei et al. (2021) reported similar biases on the perceived orientation of a search target as a function of the previous trial target and current distractors. However, those original results could reflect the fact that observers report their search template instead of the search target. Our current results suggest that this is unlikely, however. The biases created by the search task affect neutral items, and reporting the search template instead of the neutral item would make little sense in this scenario. Search templates may nevertheless play a mediating role in the observed biases (see below).

Second, the to-be-ignored items induce a bias acting in parallel with positive biases induced by attended items. The latter is often described as serial dependence and is assumed to stabilize and preserve continuity in perception in the spirit of the continuity field proposed by Fischer and Whitney (2014). Serial dependence is thought to help us deal with familiar conditions by ignoring minor changes in already perceived items and maintaining continuity in perception over time (Cicchini & Kristjánsson, 2015; Liberman, Zhang, & Whitney, 2016).

Pascucci et al. (2019) argued that perception is at any moment shaped by two contrasting history-based forces: (1) sensory adaptation, as in classic after-effects such as the tilt or motion after-effects (Gibson, 1937; Wohlgemuth, 1911), and (2) past decisions. According to their account, repulsive forces (such as those seen in various low-level negative after-effects) push perception away from recently perceived stimuli. Conversely, attractive forces dominate human perception during sequences of perceptual decisions, biasing the present sensory input so that it appears more similar to past visual input than it actually is, serving as compensation for sensory adaptation. This mechanism might explain the repulsive biases we observed. However, this similarity effect (similar distractors create attractive biases and dissimilar ones create repulsive biases) does not fit the typical pattern of sensory adaptation (stronger repulsive biases for similar inducers and weak, often attractive, or no biases for dissimilar ones; see reviews in Clifford, 2014). Note, however, that Solomon, Felisberti, and Morgan (2004) observed a pattern of results that is more similar to what we found. This explanation can nevertheless be tested in future research into the effects of different roles that items play in this interdependence.

We speculate that our findings may be related to what has been referred to as tuning of target templates through the history of both distractors (Chetverikov et al., 2020a; Geng, Won, & Carlisle, 2019) and targets (Hansmann-Roth, Thorsteinsdóttir, Geng, & Kristjánsson, 2020b; Manassi, Kristjánsson & Whitney, 2019; for a review, see Geng & Witkowski, 2019; for evidence of context-based serial dependence, see Fischer et al., 2020). Visual search templates can be optimally tuned through perceptual history to help us find items similar to the target. As Bravo and Farid (2016) put it: “Rather than being a faithful, unbiased representation of the target, the target template is a biased representation that reflects the information necessary to perform the search task.” They argued that the template is adapted to the task at hand (see also Navalpakkam & Itti, 2007), and we propose that recent perceptual history plays a crucial role in determining this bias. The representations (or templates) are dynamic, dependent on the context, and our current findings may cast light on how the templates are biased. Importantly, our results suggest that the search templates can bias perceptual decisions of irrelevant items and that these biases serve the purpose of making the objects of interest in each case more salient (assuming that the biases can influence relatively early visual processing so identifying items matching the biased search templates becomes easier during later processing).Manassi et al. (2019) reported interesting findings with respect to this in a visual classification task. They found that visual classification of single objects was serially dependent, biasing classification toward previously perceived objects, but only between similar objects and within a limited spatial window, showing the three characteristics proposed for continuity fields (featural, temporal, and spatial tuning). We speculate that this reflects the biasing of templates. The intriguing question is, therefore, whether parallel template biases can be found for distractor-based repetition effects.

### Effects of attention and proximity in feature space

In Experiment 1, where feature space distances between test line orientation and the target, on the one hand, and target orientation and distractor orientation, on the other, were selected randomly, the target caused attractive biases while there were hints of a repulsive bias from distractors. Experiments 2 and 3 then indicated that feature space proximity plays a crucial role in determining bias direction. In Experiment 2, where target orientation was close to the test line orientation, the targets caused attractive biases, but, when the same targets in Experiment 3 were far from the test line, there was no significant bias. Conversely, the distractors produced a repulsive bias upon perceived test line orientation when they were far from each other in feature space (Experiment 2) but produced an attractive bias when they were close to the test line orientation in feature space (Experiment 3). Thus, even though the distractors and targets roles in Experiment 3 were the same as in Experiment 2, a change in how similar they are to the test item affected the direction and strength of the biases. This shows an interactive relationship between feature space proximity and whether items are attended targets or distractors to be ignored.

Bliss et al. (2017) (see also Fritsche et al., 2017; Samaha et al., 2019) reported attractive biases upon orientation estimations when preceding stimuli had orientations similar to the current ones in a serial dependence paradigm involving an inducer and a test stimulus. Additionally, Fritsche et al. (2017) reported repulsive biases when the inducer and the test were dissimilar. Later, Fritsche and de Lange (2019) found that the attractive bias was strongly reduced when observers attended to a different feature of the previous stimulus than orientation, and they argued for a role of feature-based attention in determining perceptual biases. This is similar to previous findings suggesting that serial dependence is gated by attention (Fischer & Whitney, 2014; Fornaciai & Park, 2018; Liberman, Zhang, & Whitney, 2016). In contrast, repulsive biases in Fritsche and de Lange (2019) were not affected by feature-based attention. Our results partly agree with these findings but, in other ways, go against them. As in Fritsche and de Lange (2019), we found attractive biases from items similar to the test and repulsive biases from items dissimilar from the test. Furthermore, we also found that attention strengthens the attractive biases from similar items; however, in our experiments, the repulsive biases were not observed for dissimilar targets, only for dissimilar distractors. Additionally, Experiment 4 suggests that the bias from distractors is weakened when they are not directly a part of the task. In sum, our findings suggest that both attractive and repulsive biases are affected by attention but in different ways.

### Context effects and ensembles

Previous results have revealed strong effects upon response times in visual search (for a recent review, see Kristjánsson & Ásgeirsson, 2019), from both targets (Maljkovic & Nakayama, 1994) and distractors (Kristjánsson & Driver, 2008; Saevarsson, Jóelsdóttir, Hjaltason, & Kristjánsson, 2008). The current results add a crucial component to such visual search effects in showing how they affect perceptual decisions of a task-irrelevant item. Although we speculate that similar mechanisms facilitate search and cause the perceptual biases we see here, mapping their connection requires further research.

Our results also add to our understanding of these processes by demonstrating how both attended items and items that need to be ignored influence perceptual decisions. The distractor effect here is interesting in light of the finding that perception of a visual ensemble (e.g., a set of Gabor patches) is sequentially dependent on previously perceived ensembles (Manassi et al., 2017; for related findings, see Pascucci et al., 2019, Experiment 7). Our current findings reinforce this, suggesting that not only attended but also distracting ensembles create perceptual biases.

### Potential relations with visual working memory

Whether serial dependence reflects working memory function is hotly debated (for reviews, see, e.g., Kiyonaga et al., 2017; Lorenc, Mallet, & Lewis-Peacock, 2021). Interestingly, Rademaker, Bloem, De Weerd, and Sack (2015) showed that when observers have to remember the first of two sequentially presented Gabor patches, the remembered orientation of the Gabor was biased toward the second irrelevant stimulus. Similar to our conclusions here, Rademaker et al. argued that both attended and ignored information (in their case, in working memory) is used to maintain continuity within the visual environment. Golomb (2015) found that, for two simultaneously presented stimuli, memory is biased away from a distractor when it is similar to the test item but toward it when it is dissimilar (see also Bae & Luck, 2019; Chunharas et al., 2019). What is interesting about these findings is how feature space and attentional role are both critical for the biases of the representations as is the main finding here.

### Serial dependence as a general feature of perceptual mechanisms?

The wide-ranging spectrum of findings on serial dependence effects that we scratch the surface of here raises the intriguing question of whether serial dependence is a general characteristic of perceptual mechanisms or whether there is a specific mechanism devoted to promoting serial dependence. Serial dependence is unlikely to solely reflect low-level activity. For example, areas of the prefrontal cortex show activity modulations from serial dependence in working memory (Barbosa et al., 2020, although there is also evidence for serial dependence in earlier visual areas (John-Saaltink et al., 2016; van Bergen & Jehee, 2019). Cicchini, Benedetto, and Burr (2021) have recently proposed that the priors that presumably play a crucial role in serial dependence arise in higher level visual processing, propagating information down to earlier sensory processing levels. This interesting possibility invites speculation that the detailed characteristics of serial dependence may differ depending on particular circumstances—for example, whether the effects are positive or negative, large or small (for similar speculation, see Murai & Whitney, 2021). Also, their temporal profiles may differ depending on the network involved in analyzing particular aspects that serial dependence is seen for. Our results are consistent with this general scenario as they show how two separate aspects, feature proximity and attention, lead to serial dependence. In sum, serial dependence might be a general characteristic of perceptual processing at different levels of the cognitive hierarchy, from low-level sensory processing to higher level decision-making. A similar proposal regarding the nature of potentially related history effects (attentional priming) has recently been made (Kristjánsson & Ásgeirsson, 2019).

## Summary and conclusions

The most important result here is that visual search can induce biases in the perceived orientation of a test line that is unrelated to the search task. Our results also indicate that these biases are strongly determined by both attention and similarity between the search stimuli and the test item. Overall, we speculate that our results provide a glimpse of the bag of tricks that the visual system uses to optimize perceptual decisions over time. These tricks may be diverse, depending on the context, and may not always follow simple operational principles but can be highly task dependent. Biases from previous stimuli may be a general feature of perceptual mechanisms, and their diverse manifestations may reflect the operational characteristics of the particular neural mechanisms involved in each case.

## Supplementary Material

Supplement 1

## Footnotes

1 Note that we choose to remain neutral at this point on the question of whether serial dependence causes biases upon perception or decisional processes, or both. The observed bias in perceptual decisions could reflect a change in appearance or decision but a direct measure of this is not available in the present work. We therefore use the term *perceptual decisions*. Broadly defined, perceptual decision-making involves using sensory information from the environment to guide behavior.
