# Supplementary material for: You see what you look for: Targets and distractors in visual search can cause opposing serial dependencies
Source: J Vis. 2021 Sep 1;21(10):3. doi: 10.1167/jov.21.10.3 (PMC8419872; doi:10.1167/jov.21.10.3)
Supplement: Supplement 1 [file jovi-21-10-3_s001.docx]

# Supplementary information

### The role of proximity in feature space on the biases strongness

We performed an exploratory analysis to test how the distance in feature space between the test line to distractor and test line to target can affect the strength of the biases. In the following plots the x-axis shows the distance in feature space and the y-axis represents the adjustment error (biases).


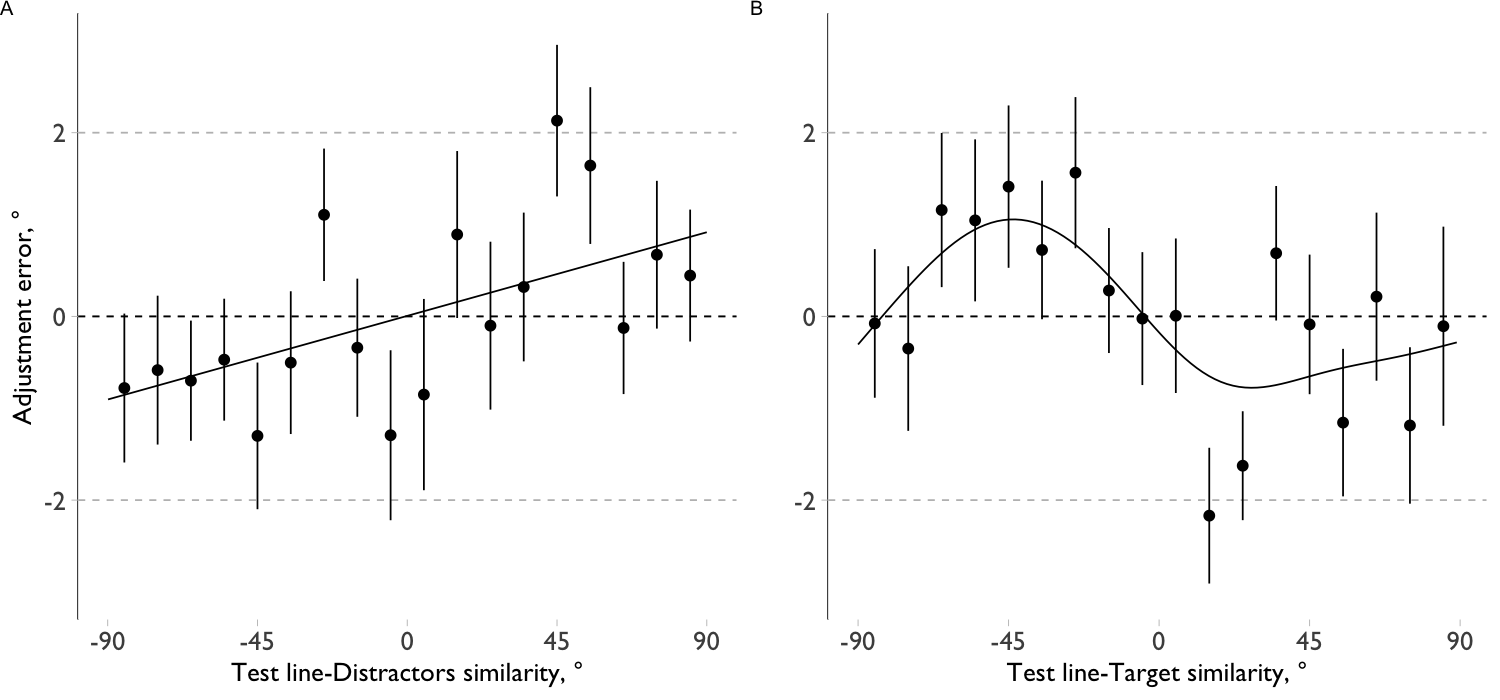


**Supplementary Fig.1.** The effect of target and distractors to test line distances in feature space on test line perception in Exp.1. The above exploratory analysis suggests that the target's attractive bias is strong when they are close to the test line orientation in feature space, and the distractor effect is in the strongest state when it is far away from the test line orientation in feature space.

### Model comparison (model with and without uniform component)

To assess whether the use of the Zhang & Luck model (mixture of Gaussian and uniform) is justified, we compared the model including the uniform component to the model that did not have it. In all cases, the model with the uniform component provided a better fit. Next, to test how much the results depend on selecting a particular model, we repeated the analyses using a simple repeated-measurements ANOVA where the adjustment error was the dependent variable and the distractor to test line conditions (distractors oriented clockwise or counterclockwise relative to the Test lines) and the target to test line conditions (Targets oriented clockwise or counterclockwise relative to the Test lines) were the independent variables. The results in all experiments were in the same direction. The only difference with respect to the results reported in the paper was that in the last experiment, the effect of distractors did not reach significance, which was expected given the lower sensitivity of this approach as this model is not the best description of the data (indicated by more imperfect fits of the model with a uniform component). The results of the repeated-measurements ANOVA are shown in Supplementary Table 1.

|  | Bias produced by distractors | | Bias produced by targets | |
| --- | --- | --- | --- | --- |
|  | *F*(1,19) | *P* | *F*(1,19) | *p* |
| Exp.1 | 1.01 | 0.328 | 24.13 | < 0.001 |
| Exp.2 | 4.68 | 0.043 | 38.09 | < 0.001 |
| Exp.3 | 12.21 | 0.002 | 2.53 | 0.128 |
| Exp.4 | 1.14 | 0.299 | 42.21 | < 0.001 |

**Supplementary Table.1.** The results of repeated-measurements ANOVAs for the biases reported in the paper without uniform component.

### Accuracy and RT in Experiments 1-4

Supplementary Table 2 presents information about the average accuracy and RT in the main tasks in Experiments 1-4. It is essential to mention that the participants were not the same in all of the experiments, and the task in Exp. 4 was not a visual search task since the locations of the targets were cued already, hence any direct comparison is unwarranted.

|  | Exp.1 | Exp.2 | Exp.3 | Exp.4 |
| --- | --- | --- | --- | --- |
| RT (milliseconds) | 896 | 731 | 729 | 621 |
| Accuracy (%) | 94 | 93 | 93 | 92 |

**Supplementary Table 2.** The accuracy and RT of the Experiments 1-4.

### The role of test line location on the biases produced by target and distractors

We performed an exploratory analysis to see if the location of the test line (whether it is presented on the search target location or the distractors' location) can alter the amplitude of the biases produced by distractors and target in perceptual decisions related to the test line orientation. We used several repeated-measurements ANOVAs where the location of the test line, distractor to test line conditions (Distractors oriented clockwise or counterclockwise relative to the Test lines), and target to test line conditions (Targets oriented clockwise or counterclockwise relative to the Test lines) were independent variables, and adjustment error was the dependent variable. In general, the biases were stronger when the test line was shown in the target location. The results are shown in Supplementary Table 3.

|  | Interaction between test line location and the biases produced by target | Interaction between test line location and the biases produced by distractors |
| --- | --- | --- |
| Experiment 1 | F(1, 19) = 0.63, p = 0.436 | F(1, 19) = 4.52, p = 0.047 |
| Experiment 2 | F(1, 19) = 0.29, p = 0.590 | F(1, 19) = 4.83, p = 0.040 |
| Experiment 3 | F(1, 19) = 6.43, p = 0.020 | F(1, 19) = 0.01, p = 0.937 |
| Experiment 4 | F(1, 19) = 4.01, p_= 0.059 | F(1, 19) = 0.04, p = 0.842 |

**Supplementary Table 3.**  The role of test line location on the biases produced by distractors and targets.
